# Atypical esthesioneuroblastoma invading oral cavity: a case report and review of the literature

**DOI:** 10.1186/1746-1596-9-10

**Published:** 2014-01-20

**Authors:** Sandra Ventorin von Zeidler, Rafaela Guidi, Rita de Cássia Gonçalves Alencar, Renato Aguiar, Elismauro Francisco Mendonça, Aline Carvalho Batista, Rejane Faria Ribeiro-Rotta

**Affiliations:** 1 Department of Pathology, Federal University of Espírito Santo, Piratininga n.180/602, Praia da Costa, Vila Velha – ES, 29101-220 Vitória, Brazil; 2 Oral Health Division, State Health Department, Goiânia, Brazil; 3 Association of Cancer Combat in Goiás, Araújo Jorge Hospital, Goiânia, Brazil; 4 School of Dentistry, Federal University of Goiás, Goiânia, Brazil

**Keywords:** Esthesioneuroblastoma, Olfactory neuroblastoma, Maxillary neoplasms, Diagnosis, Oral cavity

## Abstract

Esthesioneuroblastoma is an uncommon tumour of neuroectodermal origin. The authors describe a rare presentation of an atypical esthesioneuroblastoma invading oral cavity. The clinical presentation, aetiology, diagnosis, and management of this condition are discussed. The patient developed significant swelling in the right anterosuperior alveolar mucosa and had moderate tooth mobility. Conventional x-rays and computed tomography revealed a large osteolytic lesion, with imprecise limits. Histological findings along with immunohistochemical staining results and clinical features led to the diagnosis of high-grade esthesioneuroblastoma. Local recurrences and neck metastasis were detected. The rare oral findings produced delayed in diagnosis which may lead to a compromise in planning and execution of further radical management and thus a poor prognosis.

**Virtual slides:**

The virtual slides for this article can be found here: http://www.diagnosticpathology.diagnomx.eu/vs/1168853011139286.

## Background

Esthesioneuroblastoma (ENB) is a rare and complex tumour, representing the most undifferentiated end of the spectrum of neuroendocrine tumours. The aggressiveness of ENBs is partly due to their complex anatomical location and non-specific symptoms that lead to delay in the diagnosis. This study highlights the characteristics of clinical, histopathological and immunohistochemical features of ENB showing unusual oral involvement and its importance in the differential diagnosis of sinonasal neuroendocrine malignancies and maxillary neoplastic lesions.

ENB, also known as olfactory neuroblastoma, is an uncommon tumour of neuroectodermal origin. There remains some controversy as to the origin of ENB, although the most widely accepted opinion is that it arises from basal olfactory epithelial stem cells, which would account for the intimate relationship of most ENBs with the cribiform plate, the upper portions of the nasal septum, the superior turbinates, the lateral nasal walls, and the anterior skull base. The olfactory epithelium is composed of bipolar sensory neurons, supporting cells and basal cells that, being mitotically active, are deemed to be the ancestors of the ENB [1,2].

The non-specific symptoms of nasal obstruction, recurrent epistaxis and anosmia and the special anatomical location of the tumour often lead to a diagnosis of benign paranasal disease, delaying the correct diagnosis [1]. Intraoral findings are uncommon. This is a case report of an atypical esthesioneuroblastoma invading oral cavity.

## Case presentation

**Figure 1:**
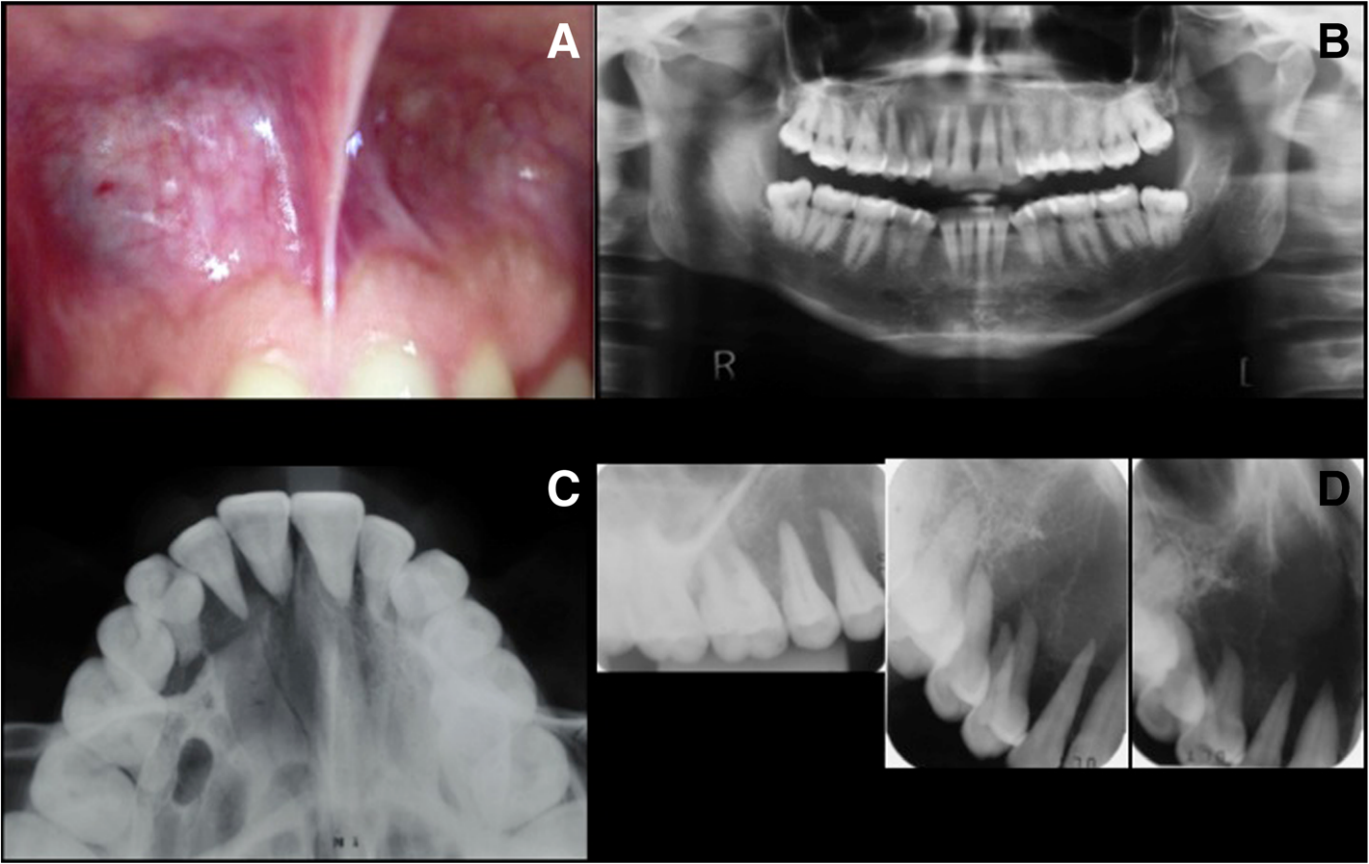
**Clinical and radiographic features. (A)** Intraoral appearance of the neoplasm at first consultation. **(B, C, D)** Panoramic, occlusal and periapical radiographs revealed a radiolucent lesion extending from the upper right first molar to the upper left canine, with imprecise limits, suggesting involvement of the floor of the nasal cavity and ipsilateral maxillary sinus. Extensive bone resorption gave the appearance of floating teeth.

A 24-year-old female was referred to an Oral Medicine Center complaining of paresthesia and tooth loosening, related to a maxillary lump, with an evolution of at least 1 year and no previous history of trauma. In the medical history of the patient there were no reports of viral infections in childhood or other systemic abnormalities. No family history of hereditary disease. She also denied any alcohol consumption or tobacco use. Cervical lymph node enlargement was not detected on palpation. Intraoral examination revealed a swelling in the right anterosuperior alveolar mucosa, which was hard on palpation, with moderate tooth mobility. The initial differential diagnosis based on clinical findings included a benign neoplasm or an odontogenic cyst (Figure 1).

**Figure 2:**
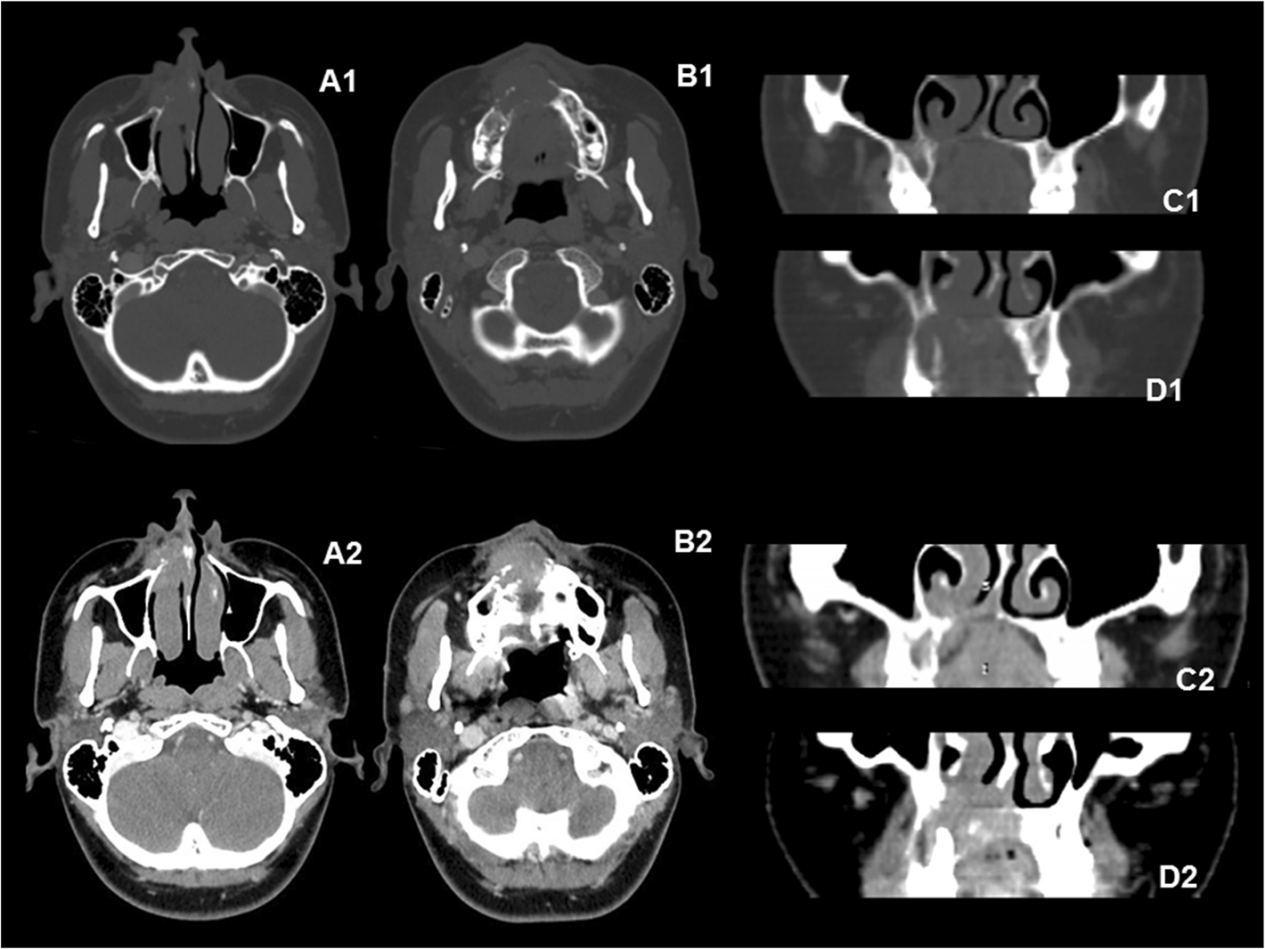
**Tomographic characteristics.** Axial scans **(A1, B1)** and coronal reformatted images **(C1, D1)** in bone window, and the same axial **(A2, B2)** and coronal **(C2, D2)** slice levels in contrast-enhanced soft tissue window. **(A, B)** The views show that the right inferior nasal concha was moderately increased in volume and tissue proliferation was observed in its anterior portion. **(C, D)** The nasal septum was partially destroyed. The lesion had invaded the alveolar process and soft tissue. Contrast enhancement was moderate and homogenous.

Conventional x-rays and computed tomography (CT) revealed osteolytic lesion with imprecise limits (± 3.0 cm) determining resorption of alveolar process and palatal bone, soft tissue invasion and floating teeth features. Superiorly, it extended into the nasal cavity, with partial destruction of the nasal septum and inferior nasal turbinate (Figures 1B-D and 2). An aspiration biopsy was negative and malignant neoplasm was considered probable diagnosis.

**Figure 3:**
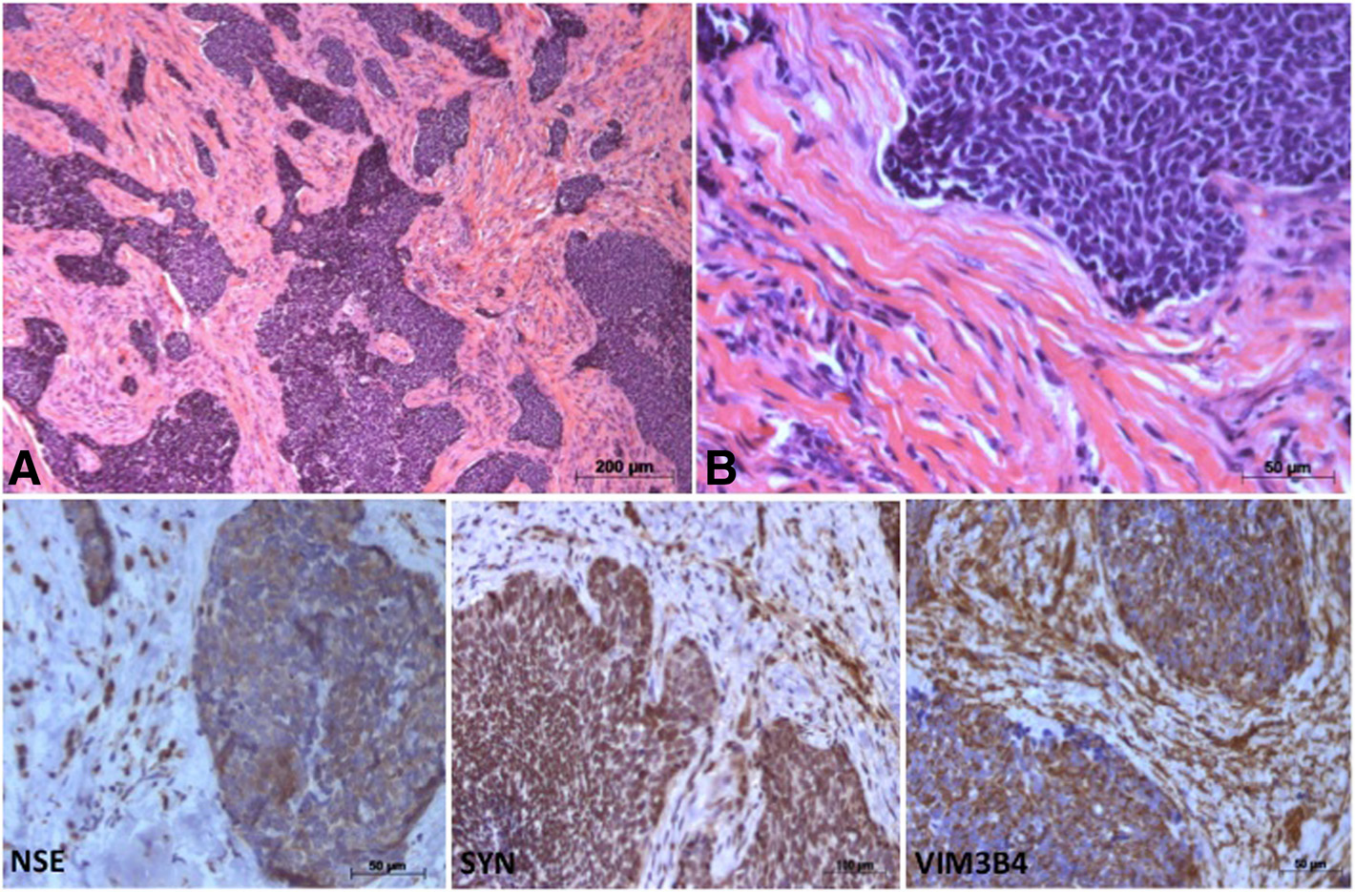
**Histopathological and immunohistochemical findings. (A)** Low-powered photomicrograph showing proliferation of islands and cords composed of small cells displaying either basaloid or fusiform morphology, a thick collagenized cellularized stroma with little inflammatory infiltrate. **(B)** In detail, neoplastic cells with pleomorphic and hyperchromatic nuclei. The cells in the tumour islands and cords were strongly positive for enolase (NSE), synaptophysin (SYN) and vimentin (VIM3B4). Some positive expression was also observed in the stromal cells. (Haematoxylin-eosin, original magnification x20[A], x100[B]; Immunohistochemical staining, original magnifications x40).

Microscopic examination of the incisional biopsy and surgical specimen revealed the proliferation of islands and cords composed of small cells displaying either basaloid or fusiform morphology. Pleomorphic and hyperchromatic nuclei with a fine granular chromatin pattern, as well as moderate mitoses and rare necrosis were observed. Fibrous connective tissue or thick collagenized cellularized stroma was visible between the islands and cords with little inflammatory infiltrate. Prominent nucleoli, rosettes of the Homer Wright type (pseudorosettes) or true rosettes were infrequent, featuring Grade III according to Hyams’ histopathologic grading system (Figure 3) [3].

The histological similarities to malignant small round cell tumours (e.g. neuroendocrine carcinoma) and basaloid squamous cell carcinoma required the use of a broad immunohistochemical panel as an adjuvant technique in the differential diagnosis. The tumour showed positive immunoreactivity to neuron-specific enolase (NSE) at 1:100 (clone BBS/NC/VI-H14, DAKO, Glostrup, Denmark), synaptophysin (Syn) at 1:200 (polyclonal, Novocastra, Newcastle, UK) and vimentin at 1:2000 (clone VIM3B4, Novocastra), along with focal positive immunoreactivity to cytokeratin at 1:800 (clone AE1/AE3, Novocastra) and carcinoembryonic antigen (CEA) at 1:2000 (polyclonal, DAKO). A few cells showed a positive reaction to S-100 protein at 1:600 (polyclonal, Novocastra), chromogranin A at 1:400 (clone LK2H10, Novocastra) and p53 at 1:100 (clone DO-7, Novocastra). Immunohistochemical staining was negative for neuroblastoma marker at 1:400 (clone NB84a, DAKO) (Figure 3). Histological findings along with immunohistochemical staining results and clinical features led to the diagnosis of high-grade esthesioneuroblastoma.

The patient underwent partial maxillectomy and immediate rehabilitation with obturator prostheses. Adjuvant radiotherapy was given over 6 weeks with a total dose of 6000 Gy. The patient presented with two recurrences 12 and 22 months after radiotherapy. In the first recurrence the patient underwent infrastructure maxillectomy. The second recurrence was diagnosed and a CT scan revealed neck metastasis. The patient refused further surgery and palliative chemotherapy was initiated.

## Discussion

Sinonasal neuroendocrine malignancies are complex and rare, with ENB representing the most undifferentiated end of the spectrum of neuroendocrine tumours. ENB accounts for approximately 3% to 6% of nasal cavity and paranasal sinus cancer cases, 0.3% of upper aerodigestive tract malignancies and less than 1% of all head and neck cancers. This tumour shows no predilection for gender and occurs over a wide age range, though a bimodal age distribution with an early peak from 11 to 20 and a later peak between 51 and 60 years of age has been reported [2,4].

ENB is characterized by slow progression and locally aggressive behaviour, which lead to long-term survival but very frequent late local recurrence. The aggressiveness of ENBs is partly due to their complex anatomical location, close to vital structures, which is associated with non-specific symptoms that lead to delay in the patient seeking diagnosis [1,5,6]. The reported case revealed an atypical ENB identified by oral symptoms, with radiographic images that suggested a maxillary neoplasm invading the nasal cavity. However, axial CT images suggested that the epicentre of the lesion was in the nasal region and that it was invading the alveolar process (Figure 2).

The diagnosis of ENB via light microscopy by itself can be difficult since the tumour tends to exhibit little or no differentiation. Pathological classification is challenging because the tumours must be differentiated from other small cell neoplasms of the nasal cavity such as Non-Hodgkin’s lymphoma, Ewing’s sarcoma, mucosal malignant melanoma and neuroendocrine carcinomas [2]. In this case, given the relation with the oral mucosa, basaloid squamous cell carcinoma was also included in the differential diagnosis.

The histopathological parameters that help in differentiating these tumours include the pattern of tumour cell arrangement, stroma, nuclear chromatin characteristics, presence or absence of neuropil and resetting. The use of a broad panel of antibodies in immunohistochemical staining may help to establish a final diagnosis. ENB is usually positive for general neuroendocrine markers, such as neuron specific enolase (NSE), S-100 protein, synaptophysin (Syn) and chromogranin. NSE is typically the most positive immunohistochemical stain in ENB. S-100 protein positive peripheral dendritic cells corresponding to Schwann cells may be present within the neoplasm or at the edges of tumour nests. Positivity varies in the cases reported in the literature for vimentin, keratin, glial fibrillary acidic protein, and neurofilaments [5,6]. In the reported case the tumour showed strong positive expression of NSE, Syn and vimentin. Significant correlation between CD44 expression and the stage of the disease has been suggested to help predicting clinical outcome. CD44s negative tumors are significantly correlated with the lack of differentiation. Thus, overexpression of CD44s could be considered as a predictor of the absence of infiltration of the tumour and neuroblastic tumors subtypes of favorable prognosis [7].

The possibility of basaloid squamous cell carcinoma and primitive neuroendocrine tumour was not supported by immunohistochemistry, and this was reinforced by the focal expression of carcinoembryonic antigen and pan-cytokeratin. The small number of labeled cells at P53 suggested a more favorable prognosis in terms of tumour proliferation [6].

The rates of primary tumour recurrence vary and most of the case series show local recurrence rates of approximately 14 to 30%. The mean time to recurrence is 2 years, but recurrences can occur as late as 10 years after the initial diagnosis, with approximately 50% of them occurring after 5 years [1,5,6,8].

Craniofacial resection with definitive or adjuvant radiotherapy has been used for local control. Chemotherapy can be used in an adjuvant/neoadjuvant attempt and also in the metastatic phase, or recurrent or advanced disease, although its effectiveness has still not been established. Such multimodality therapy has become the most common approach to ENB [4,9-12].

The Kadish staging system is a clinically based staging system for sinonasal tumors and the ENB herein described was classified as Kadish stage C (extension of tumour beyond the nasal cavity and paranasal sinuses) [9,10]. However, sectional images showed local disease in an uncommon location, the anterior part of the inferior nasal concha extending to another atypical area – the maxillary alveolar ridge. Due to the large number of neoplasms and cystic lesions affecting the jaws, extension of the tumour into the oral cavity was causing tooth mobility, mimicking a neoplasm of that region.

In this case report, the rare oral findings produced delayed in diagnosis leading to a compromise in planning and execution of adequate management. Only another two cases of ENB in the literature included intraoral findings, confirming the importance of the knowledge about different clinical presentations of ENB [13,14]. We believe that the difficulty in diagnosis and inadequate management due to atypical oral cavity localization may lead to a compromise in planning and execution of further radical management and thus a poor prognosis. This highlights the importance of the dentist’s knowledge about different clinical presentations of ENB. The two local recurrences of this reported case were also observed primarily in the oral cavity. Despite surgery and radiotherapy, cervical metastasis was detected 12 months after the end of treatment. Based on the histological grading, the tumor of this patient can be categorized as high-grade malignancy and along with advanced clinical stage (Kadish C) may be associated with locoregional recurrence and clinical spread of tumor.

## Conclusions

In conclusion, this study highlights the characteristics of clinical features of ENB showing oral involvement and its importance in the differential diagnosis of sinonasal neuroendocrine malignancies. We believe this report has value as another atypical case of ENB and also has teaching value for training in the differential diagnosis of maxillary neoplastic lesions.

## Consent

Written informed consent was obtained from the patient for publication of this Case Report and any accompanying images. A copy of the written consent is available for review by the Editor-in-Chief of this journal.

## Abbreviations

ENB: Esthesioneuroblastoma; CT: Computed tomography; NSE: Neuron-specific enolase; Syn: Synaptophysin; CEA: Carcinoembryonic antigen.

## Competing interests

The authors declare that they have no competing interests.

## Authors’ contributions

SVVZ designed the study and wrote the paper; RG, EFM and RA participated in providing the clinical information of this case; EFM, RCGA and ACB performed the histological evaluation and carried out the immunoassays; RFRR was involved in literature search and preparing the material. All authors read and approved the final manuscript.
